# Cytokeratin-positive cells in the bone marrow from patients with pancreatic, periampullary malignancy and benign pancreatic disease show no prognostic information

**DOI:** 10.1186/s12885-020-07510-z

**Published:** 2020-11-16

**Authors:** Harald Hugenschmidt, Knut Jørgen Labori, Cathrine Brunborg, Caroline Sophie Verbeke, Lars Thomas Seeberg, Cecilie Bendigtsen Schirmer, Anne Renolen, Elin Borgen, Bjørn Naume, Gro Wiedswang

**Affiliations:** 1 Institute of Clinical Medicine, University of Oslo, Oslo, Norway; 2 Department of Transplantation Surgery, Oslo University Hospital, PO.Box 4950, NO-0424 Oslo, Nydalen Norway; 3 Department of Gastrointestinal Surgery, Oslo University Hospital, Oslo, Norway; 4 Oslo Centre for Biostatistics and Epidemiology, Oslo University Hospital, Oslo, Norway; 5 Department of Pathology, Oslo University Hospital, Oslo, Norway; 6 Department of Gastrointestinal Surgery, Vestfold Hospital Trust, Tønsberg, Norway; 7 Deparment of Oncology, Oslo University Hospital, Oslo, Norway

**Keywords:** DTC, Disseminated tumour cells, Bone marrow, Pancreatic cancer, Periampullary cancer

## Abstract

**Background:**

Pancreatic and periampullary carcinoma are aggressive tumours where preoperative assessment is challenging. Disseminated tumour cells (DTC) in the bone marrow (BM) are associated with impaired prognosis in a variety of epithelial cancers. In a cohort of patients with presumed resectable pancreatic and periampullary carcinoma, we evaluated the frequency and the potential prognostic impact of the preoperative presence of DTC, defined as cytokeratin-positive cells detected by immunocytochemistry (ICC).

**Methods:**

Preoperative BM samples from 242 patients selected for surgical resection of presumed resectable pancreatic and periampullary carcinoma from 09/2009 to 12/2014, were analysed for presence of CK-positive cells by ICC. The median observation time was 21.5 months. Overall survival (OS) and disease-free survival (DFS) were calculated by Kaplan-Meier and Cox regression analysis.

**Results:**

Successful resections of malignant tumours were performed in 179 of the cases, 30 patients resected had benign pancreatic disease based on postoperative histology, and 33 were deemed inoperable intraoperatively due to advanced disease. Overall survival for patients with resected carcinoma was 21.1 months (95% CI: 18.0–24.1), for those with benign disease OS was 101 months (95% CI: 69.4–132) and for those with advanced disease OS was 8.8 months (95% CI: 4.3–13.3). The proportion of patients with detected CK-positive cells was 6/168 (3.6%) in resected malignant cases, 2/31 (6.5%) in advanced disease and 4/29 (13.8%) in benign disease. The presence of CK-positive cells was not correlated to OS or DFS, neither in the entire cohort nor in the subgroup negative for circulating tumour cells (CTC).

**Conclusions:**

The results indicate that CK-positive cells may be present in both patients with malignant and benign diseases of the pancreas. Detection of CK-positive cells was not associated with differences in prognosis for the entire cohort or any of the subgroups analysed.

**Trial registration:**

clinicaltrials.gov (NCT01919151).

## Background

According to their anatomical origin, the main carcinoma types of the periampullary region are pancreatic ductal adenocarcinoma (PDAC), distal bile duct cancer (DBDC), ampullary cancer (AMPUC), and duodenal cancer (DUODC). They constitute an entity of aggressive tumours, particularly PDAC and DBDC where 80% of the cases have either locally advanced or metastatic disease at the time of diagnosis [1, 2]. These tumours are potentially curable when diagnosed at an early, localised stage [3]. Still, the results from state of the art “surgery first” strategy [4], with radical resection followed by adjuvant therapy, are dismal. The expected rate of relapse during the first year after surgical resection is 50% and 5-year survival rates below 10% [1, 3].

The BM is a known reservoir for dormant or slowly proliferating, disseminated tumour cells (DTCs) [5], being part of the metastatic cascade in malignancies of epithelial origin [6]. The best validated DTC-detection method for BM-samples is based on density centrifugation and immunocytochemical (ICC) identification of a panel of Cytokeratins (CK). The morphological evaluation and categorization of the cells follows internationally standardized criteria by the ISHAGE-group [7–9]. Beyond prognostic information, there is well established evidence for the clinical utility of DTC detection in the management of the early stages of both breast [10, 11] and prostate cancer [5, 12]. There are several reports on the impact of DTC in pancreatic cancer in general, indicating an association of DTC with reduced survival [13–17]. However, for the potential application as a predictive tool for presumed resectable periampullary cancers, the clinical relevance of DTC is unclear. A recent meta-analysis exploring the significance of CTC and DTC in PDAC-patients [18] revealed a significant association between DTC and survival for the entire cohort containing a mix of resectable and advanced cancers. In the subgroup of resected cancers only, the same study did not disclose a significant association, even though another study [13] had reported an association between DTC and impaired survival previously.

The aim of the present study was to explore the frequency and the prognostic impact of preoperatively detected DTCs in a cohort of patients with presumed resectable pancreatic and other periampullary cancers.

## Methods

### Patients, study design and follow-up

Between September 2009 and December 2014, patients referred to Oslo University Hospital, Norway with potentially resectable pancreatic and periampullary malignancy were included in a standardised preoperative workup and evaluation in a multidisciplinary tumour board as detailed previously [19]. Clinical data were recorded prospectively in an Epi-Info 3.5.3 database (CDC; Atlanta, GA; USA). The follow up included clinical status, computed tomography (CT) of the chest and abdomen as well as CA19–9 assessment twice a year. The mortality was deducted from the Norwegian Cause of Death Registry, provided by the Norwegian Institute of Public Health. The same patient cohort was described previously [20], with the observation period further extended by 25 months to January, 31st 2019. The study was undertaken in accordance to the STROBE(2014) and REMARK(2012) criteria for analysis and reporting.

### Detection of CK-positive cells in the bone marrow

BM samples were collected under anaesthesia just prior to surgery, five ml BM from the anterior iliac crest bilaterally in syringes containing 200 IE Heparin in 0.5 ml NaCl. Samples were processed within 24 h at the Micrometastasis Laboratory, Oslo University Hospital, Norway as previously described [21]. BM mononuclear cells were isolated by density centrifugation over a Ficoll-Hypaque gradient (Lymphoprep®, Abbott Rapid Diagnostics AS, Oslo Norway) and cytospin-slides were prepared with 0.5 × 10^6^ BM-MNC/slide. For each sample 4 slides (i.e. 2 × 10^6^ cells) were immunostained for CK-positive cells with a combination of the monoclonal mouse primary antibodies AE1 and AE3 (Prod.# MAB 1612 & 1611, Milipore), with a broad affinity for the acidic and basic cytokeratin types, namely CK 1–8, 10, 14–16 and 19. The APAAP method for detection used a rabbit anti-mouse secondary antibody (Dako, #Z0259) and an alkaline phosphatase-mouse-anti-alkaline phosphatase tertiary antibody (Dako, #D0651), followed by a colour reaction with New Fuchsin, staining positive cells red [22]. Nuclear counterstaining was performed with haematoxylin. The cytospins were screened for ICC-positive cells in an automated Ariol SL50 analyser (Leica biosystems). Detected elements were reviewed by a trained research engineer (CBS) and candidate cells were classified by a dedicated pathologist (EB). The cytomorphological evaluation of detected ICC-positive cells was performed in accordance to the ISHAGE consensus guidelines [7, 8]. This protocol, originally validated for breast cancer samples, has become the de facto standard for DTC reporting, including pancreatic cancer studies [13]. Based on this classification, and according to earlier practice [23], the ICC-positive cells are divided into 4 categories: tumour cells (TC), hematopoietic (ie “false positive”) cells (HC), QHC (questionable/probable HC) and uninterpretable cells (UIC). If cells classified as TC or UIC were detected, 4 additional cytospins were incubated with the non-reactive mouse monoclonal antibody MOPC21 (Prod# M9269, Sigma Aldrich) of the same Ig isotype as AE1AE3, and detected by APAAP as above, constituting a negative control for the ICC reaction. Samples harbouring cells classified as TC in AE1/AE3 slides and not in the corresponding negative control slides were classified as “DTC-positive”. Samples harbouring cells classified as TC in AE1/AE3 slides and in the corresponding negative control slides were interpreted as “not evaluable” (n.e.) and excluded from further analysis. Samples harbouring “UIC”, “HC” or “QHC” were interpreted as “DTC-negative”. Results were stored in a database at the Micrometastasis Laboratory, Dept. of Pathology, Oslo University Hospital and were not available to treating clinicians. Following closure of the observation period, the classification of ICC-positive cells was combined with the clinical data using a scripted tool upon import to SPSS.

### Characteristics of the patient cohort

**Fig. 1:**
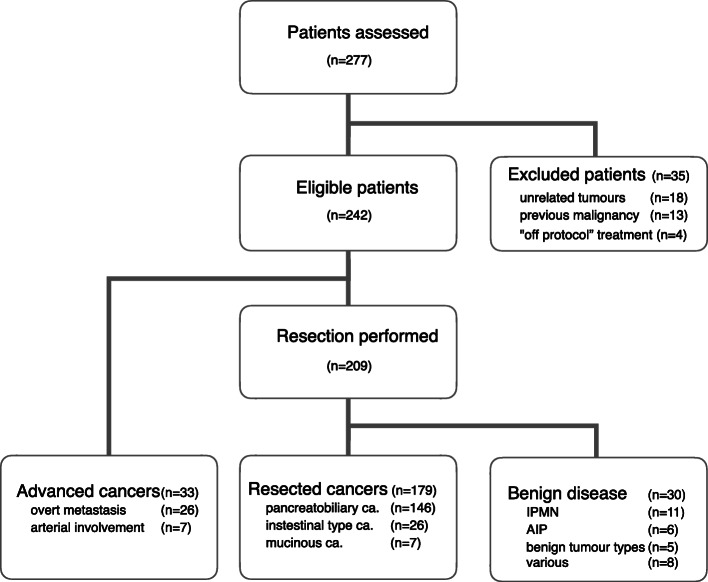
Stratification of the patient cohort Legend: Study overview, showing group distribution, specifying causes for exclusion, histologic types, reasons for advanced cancer status and benign diagnoses. AIP: autoimmune pancreatitis; IPMN: intraductal mucinous neoplasia

Patients were categorised into three distinct clinical groups, see also Fig. 1. Patients who underwent surgical resection did either have confirmed malignancy (resectable cancer) or a non-malignant condition (benign disease). The third group consisted of advanced cancer patients who underwent exploratory laparotomy but did not undergo resection, either due to locally advanced tumour growth or overt metastases (advanced cancer).

In addition to the presence of ICC-positive cells, the following clinical- and pathological parameters were recorded: age, gender, CA19–9, tumour size on CT-scan, AJCC/UICC-stage (7th ed.), pTNM-staging including resection margin, cancer origin, grade of histopathological differentiation, histological subtype (predominantly intestinal or pancreatobiliary), vascular and perineural infiltration. Continuous variables were dichotomized at the following thresholds: CA19–9 ≥ 200kU/L and the size of the lesion on CT-scan ≥25 mm (for results, see Table 1).

**Table 1:** Clinical characteristics of the cohort according to patient groups including ICC-positive cell type status in bone marrow

	Resected cancers(*n* = 179)	Advanced cancers(*n* = 33)	Benign(*n* = 30)
Age, median [years]	69 (34–88)	63 (46–83)	69 (45–82)
Sex, male	92 (51.4%)	19 (57.6%)	21 (70.0%)
ICC-positivity: ≥ 1 ICC-positive cell / 2 × 10^6^ MNC			
(ICC-status numbers may differ due to varying number of inconclusive ICC-results per category)			
TC	6/168 (3.6%)	2/31 (6.5%)	4/29 (13.8%)
UIC	10/174 (5.7%)	0/31 (0.0%)	2/30 (6.7%)
HC	16/169 (9.5%)	2/30 (6.7%)	3/29 (10.3%)
QHC	12/135 (8.8%)	3/28 (7.9%)	3/25 (12.0%).
Preoperative Risk Factors			
CA19–9 ≥ 200kU/l	51/127 (40.2%); *52 missing*	17/28 (60.7%); *5 missing*	0/20; *10 missing*
Tumour size> 25 mm	71 (39.7%)	20 (60.6%)	10 (33.3%)
Bilirubin > 50 μmol/L	132/151 (87.4%); *28 missing*	21/32 (65.6%); *1 missing*	7/29 (24.1%); *1 missing*
Treatment			
Neoadjuvant chemotherapy	**GEMZ** 6 (4.1%) **FOLFIRINX** 1 (0.7%)	**GEMZ** 1 (3.0%)	none
Operation			
PPPD	146 (82.6%)	**BDB** 24 (72.7%)	29 (96.7%)
PD	25 (14.0%)	**Exp**.**lap.** 9 (27.3%)	1 (3.3%)
Pancreatectomy	8 (4.5%)		none
Venous resection	49 (27.4%)	n.a.	2 (6.7%)
Adjuvant chemotherapy	**FLV**: 91 (50.8%)**GEMZ**: 7 (3.9%)**FLOX**: 1 (0.7%)**none:** 80 (44.7%)	Palliative chemotherapy31 (93.9%)	n.a.
Histopathologic results			
Pancreatic cancer	101 (56.4%)	30	
Malignant IPMN	9 (5.0%)		
Distal bile duct ca.	31 (17.3%)	1	
Ampullary cancer	21 (11.7%)		
Duodenal cancer	17 (9.4%)	2	
Pancreatobiliary type	146 (81.6%)	28 (84.8%)	**IPMN** 11 (36.7%)
Intestinal type	26 (14.5%)	5 (15.2%)	**ben.Tu.** 5 (16.6%)
Mucinous type	7 (3.9%)		**AIP** 6 (20.0%)
			**chr.Panc** 4 (13.3%)**inflam.** 4 (13.4%)
UICC-stage (V7): I	16 (10.6%)		
II	150 (83.8%)		
III	13 (7.3%)	7 (21.2%)	
IV		26 (78.8%)	
N1-status	120 (67.0%)		
R1-status	87 (48.6%)		
Vascular infiltration	108 (60.3%)		
Perineural infiltration	141 (78.8%)		

*GEMZ* Gemzitabine, *FLV, FLOX, FOLFIRINOX* Chemotherapy regimens; *PD* Pancreatico-duodenectomy, *PPPD* Pylorus preserving PD, *BDB* Biliodigestive bypass; *Ex.lap*. Explorative laparotomy; *IPMN* Intraductal pancreatic mucinous neoplasia; *ben.tu*. Benign tumour; *AIP* Autoimmune pancreatitis; *chr. Panc*. Chronic pancreatitis; *inflam*. Inflammation *n.a*. Not applicable; *n.d*. Not determined. Note: ICC-status numbers may differ due to varying number of inconclusive ICC-results per category

### Statistics

Data were analysed in IBM SPSS, V25 (IBM Cooperation Analytics, Armonk, NY, USA) and STATA 15 (Stata Corp LLC, College Station, Texas, USA). Graphs were prepared in PRISM 8 (GraphPad Software Inc., La Jolla, CA, USA). The primary endpoints of the study were overall survival (OS), defined as survival until death by any cause and disease-free survival (DFS), defined as survival until signs of local relapse or metastasis were detected. Survival analyses were carried out with the Kaplan-Meier method, using the Log-rank test for difference of curve pairs. The association between TC-status and survival was quantified by a hazard ratio (HR) with a 95% confidence interval (CI) using Cox regression analysis. Statistical significance was assumed for *p* < 0.05.

## Results

### Patient group characteristics

Two hundred seventy-seven patients were assessed during the study period of whom 35 patients (12.6%) did not meet the inclusion criteria. Tumour resection with curative intent was performed in 86.4% of the patients (209/242), while in 13.6% (33/242) resection was not performed due to intraoperative detection of advanced disease. In patients deemed unresectable intraoperatively, a biliary or intestinal bypass procedure was performed in 24 patients and 9 received an explorative laparotomy. Of the cases with advanced disease, the tumour origin was the pancreatic head in 30 cases, the duodenum in two cases and in one case the distal bile duct. Of the 209 cases where a successful resection was performed, malignant tumours were confirmed in 85.6% (179/209), while 14.4% (30/209) a benign pancreatic disease was found. Thus, for the whole cohort, resected malignancies account for 74.0% (179/242), advanced disease 13.6% (33/242) and benign pancreatic disease for 12.4% (30/242). The 90-day mortality in operated patients was 2.1% (5/242), all due to rapid cancer progression because of advanced disease, in one case additionally complicated by an anastomotic failure. 84.6% (31/212) of all patients with malignant disease were alive at the date of last follow-up. Ten patients had died from other causes than cancer relapse. Further details on clinical features, distribution of procedures and histopathological diagnoses of the cohort are summarised in Table 1.

### Overall survival for patient groups

**Fig. 2:**
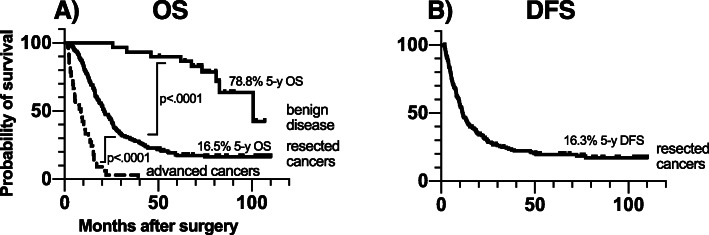
Overall- and disease free survival for patient groups. Legend Kaplan-Meier curves, with 5-year survival and *P* values (log Rank) for pairwise comparison between patient groups. A, Overall survival. B, Disease-free survival for resected patients. **DFS:** disease-free survival; **OS**: overall survival

The median observation time for the entire cohort was 21.5 months (range 1.5–110). Median overall survival (OS) was estimated to 101 months (95% CI: 69.4–132) in patients with a benign lesion, 21.1 months (95% CI: 18.0–24.1) in the resectable cancer group and 8.8 months (95% CI: 4.3–13.3) in patients with advanced cancer. Differences in OS between the subgroups were statistically significant (see also Fig. 2).

### Bone marrow ICC-positive cell types and patient survival

**Fig. 3:**
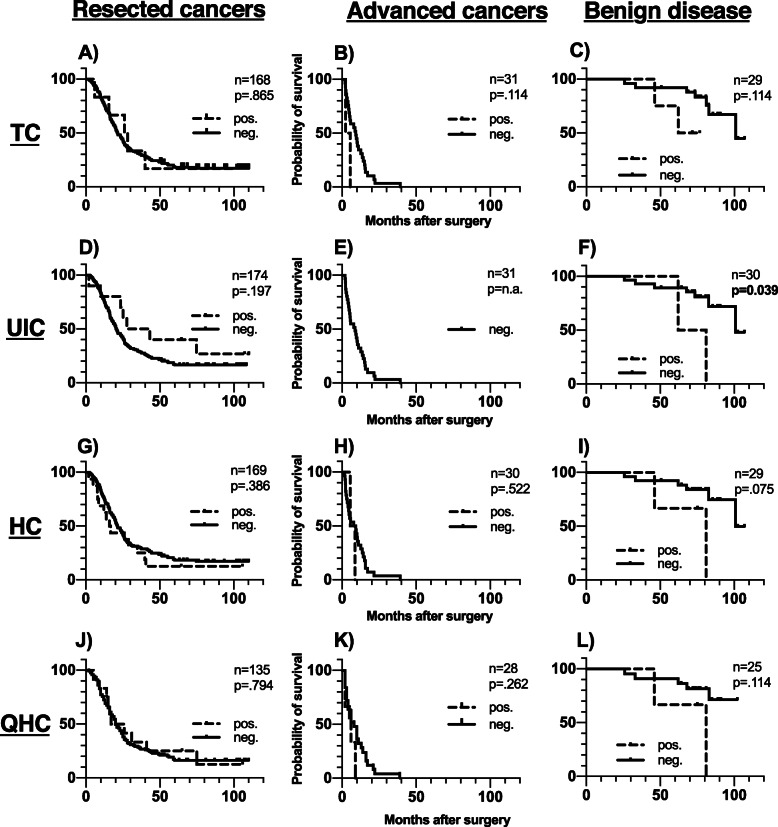
Overall survival in disease groups according to ICC-positive cell type status. Legend: Overall survival among patients with (pos.) or without (neg.) ICC-positive cells in the bone marrow within the indicated morphological cell categories. P values were computed by log-rank test, assuming *p* < 0.05 for significance. The number of cases with determined CK-status differs from group size due to cases of inconclusive ICC-results

**Fig. 4:**
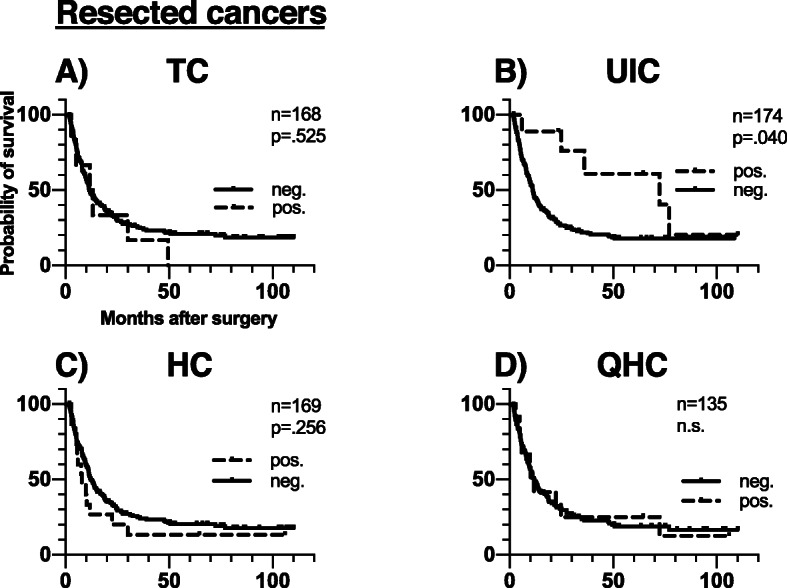
Disease free survival among resected cancers according to ICC-positive cell type status. Legend: Disease free survival among patients with (pos.) or without (neg.) CK-positive cells in the BMwithin the indicated morphological cell categories. P values were computed by log-rank test, assuming *p* < 0.05 for significance. The number of cases with determined CK-status differs from group size due to cases of inconclusive ICC-results

ICC-positive cells of the named subtypes were identified both in patients with resected malignant tumours, in those with advanced cancers as well as in patients with benign pancreatic disease. According to patient group, DTCs were present in 6/168 (3.6%) in resected cancers, 2/31 (6.5%) in advanced cancers and 4/29 (13.8%) in cases of benign pancreatic disease. The frequency of the other classes of ICC-positive cells in each prognostic group is presented in detail in Table 1. A per case specification of the distribution and frequency of all cell types together with the clinicopathological data are presented in Additional file 2. The low number of ICC-positive samples precludes subgroup analysis by tumour type. Survival analyses according to presence or absence of DTCs and the other ICC-positive cells are presented in Fig. 3 and Fig. 4, and in further detail in Table 2. DTC-positivity was associated with neither OS nor DFS. Furthermore, presence of other ICC-positive cells did not negatively impact on survival. The association between survival and presence of ICC-positive cells for benign disease was hampered by too few ICC-positive cases.

**Table 2:** Overall survival according to ICC-positive cell type status in bone marrow

ICC-type	Level	N	Incidentcases	Person months	HR(95%CI)	***p***-value	Mean OS(95%CI)
**Overall survival, resected cancers**							
**TC**	Positive	6	5	217.5	1.1 (0.4–2.3)	0.865	36.2 (11.3–61.2)
	Negative	162	133	5293.6			37.1 (31.6–42.6)
**UIC**	Positive	10	7	488.1	0.6 (0.3–1.3)	0.197	52.5 (27.2–77.7)
	Negative	164	136	5144.0			35. 6 (30.2–40.9)
**HC**	Positive	16	14	431.6	1.3 (0.7–2.2)	0.386	29.6 (14.3–44.8)
	Negative	153	125	5006.5			37.3 (31.6–43.1)
**QHC**	Positive	12	10	419.0	0.9 (.5–1.8)	0.795	37.1 (18.1–56.0)
	Negative	123	102	3817.1			35.5 (29.2–41.8)
**Overall survival, advanced cancers**							
**TC**	Positive	2	2	7.9	3.4 (0.7–15.4)	0.114	3.9 (0.8–7.1)s
	Negative	29	29	298.8			10.3 (7.4–13.2)
**UIC**	Positive	0	0		–	–	
	Negative	31	31	306.6			9.9 (7.1–12.7)
**HC**	Positive	2	2	14.3	1.6 (0.4–7.2)	0.522	7.2 (4.0–10.3)
	Negative	28	28	266.6			9.5 (6.5–12.5)
**QHC**	Positive	3	3	16.6	2.1 (0.6–7.2)	0.262	5.5 (1.9–9.2)
	Negative	25	25	248.5			9.9 (6.5–13.4)
**Overall survival, benign disease**							
**TC**	Positive	4	2	256.1	4.0 (0.7–22.6)	0.114	65.3 (53.0–77.6)
	Negative	25	7	1929.4			92.2 (82.7–101.7)
**UIC**	Positive	2	2	143.0	5.7 (1.1–25.5)	**0.039**	71.5 (52.9–90.1)
	Negative	28	7	1796.9			92.6 (83.2–101.9)
**HC**	Positive	3	2	202.3	4.7 (0.9–25.7)	0.075	69.4 (43.1–95.7)
	Negative	26	6	1867.3			93.7 (84.3–103.2)
**QHC**	Positive	3	2	202.3	3.9 (0.7–21.6)	0.114	5.5 (1.9–9.2)
	Negative	22	5	1639.1			9.9 (6.5–13.4)
**Disease free survival, resected cancers**							
**TC**	Positive	6	6	112.4	1.3 (0.6–3.0)	0.525	18.7 (4.5–33.0)
	Negative	162	126	4022.2			32.2 (25.9–38.5)
**UIC**	Positive	10	5	441.8	0.4 (0.2–1.0)	**0.040**	62.1 (37.1–87.1)
	Negative	164	132	3785.8			29.4 (23.5–35.4)
**HC**	Positive	16	16	299.4	1.4 (0.8–2.5)	0.277	22.6 (5.7–39.5)
	Negative	153	120	3774.0			31.9 (25.5–38.3)
**QHC**	Positive	12	10	340.7	1.0 (0.5–1.9)	0.973	30.1 (10. 1–50.8)
	Negative	123	96	2825.6			30.8 (23.7–37.8)

Detailed survival analysis for patients with (pos.) or without (neg.) ICC-positive cells in the bone marrow in the indicated morphological cell categories. HR and *p*-values were computed by Cox-regression analysis. The number of patients analysed may differ from group size for cases with inconclusive ICC-results

**Table 3:** Published studies on DTC status in the bone marrow for peri-ampullary adenocarcinomas, grouped by detection method

Authors	Method/ Markers	Detection limit	DTC def.	Pat. number	Loc. / Adv.	TC-rate	false-pos. Rate	Prog.	Comments
Immunocytochemistry									
Effenberger 2012 [13]	CK A45-B/B3	≥1 / 2 × 10^6^ MNC	TC	*n* = 175	*n* = 71 / *n* = 104	13.7% (24/175)	–	Pos.	29 pat. Resected despite advanced cancer stage
Rehders 2012 [25]	CK A45-B/B3	≥1 / 1 × 10^6^ MNC	TC	*n* = 49	*n* = 49 /-	27% (12/49)	–	Neg.	No details on survival analysis disclosed
Roder 1999 [17]	CK 2; KL1; A45-B7B3	≥1 / 5 × 10^5^ MNC	TC	*n* = 48	*n* = 8 / *n* = 40	52.1% (25/48)	–	Pos.	Association with OS for resected patients
van Heek 2001 [16]	CK (8, 18)	≥1 / 1 × 10^7^ MNC	TC	*n* = 35	*n* = 14 / *n* = 13	34.2% (12/35)	50%(2/4)	Pos.	2/4 benign pat. False positive
Vogel 1999 [14]	panCK, mucin, CEA, Ca-19–9	≥1 / 1.25 × 10^6^	TC	*n* = 80	*n* = 11 / *n* = 60	38%(27/71)	6.6%(3/45)	Pos.	
Thorban 1996 [15]	CK A45-B/B3	≥1 / 5 × 10^5^ MNC	TC	*n* = 42	*n* = 24 / *n* = 18	57.1% (24/42)	–	Pos.	Association with metastatic and local relapse
Z’Graggen 2001 [26]	CK AE3/AE1	≥1 / 5 × 10^6^ MNC	TC	*n* = 54	*n* = 3 / *n* = 51	24% (13/54)	4.1%(1/24)	Neg.	
Nucleotide based detection									
Hoffmann 2007 [24]	rt-PCR / CK-19	–		*n* = 37	*n* = 7 / *n* = 30	67%(25/37)	–	Neg.	
Soeth 2005 [23]	rt-PCR / CK-20	–		*n* = 117	*n* = 172	33.3%(45/135)	11%	Neg.	Survival data only for blood/BM combined. False positive rate 11%

*Prog* Prognostic impact of DTC-status on survival *Loc* Localised cancers; *Adv* Advanced cancers; *MNC* Mononuclear cells

**Fig. 5:**
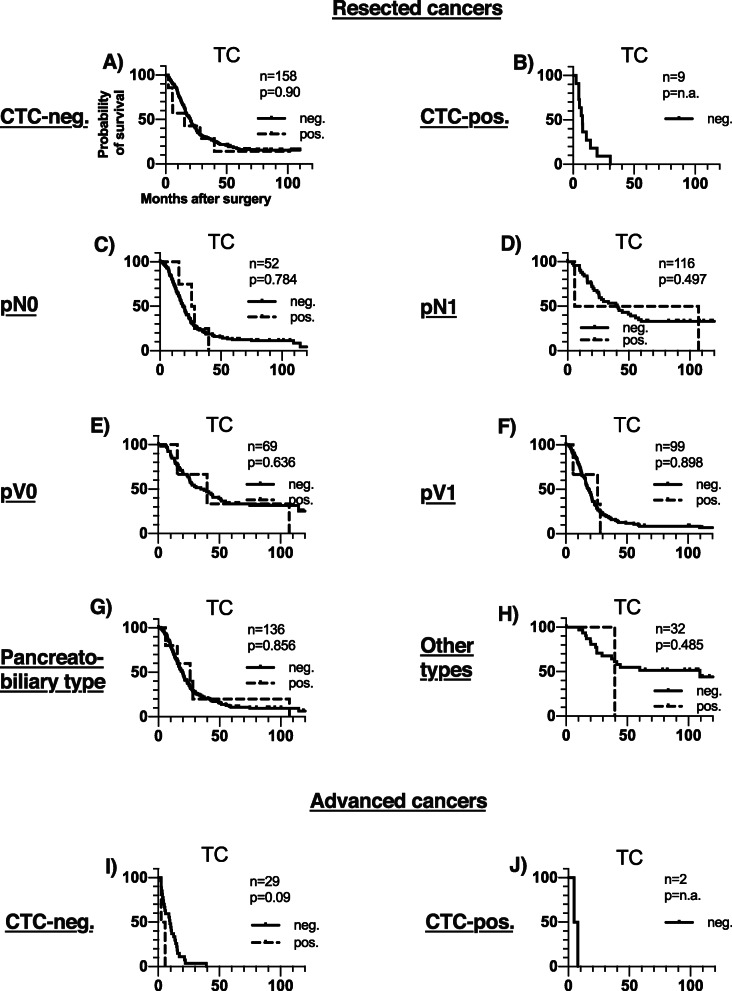
Cancer specific survival by prognostic subgroups in resected and advanced cancers according to TC-status. Legend: Overall survival dependent on CTC-status for resected and advanced cancer patients with (pos.) or without (neg.) TC cells. P values were computed by log-rank test, assuming *p* < 0.05 for significance. The number of cases with determined CK-status differs from group size due to cases of inconclusive ICC-results

To account for the effect of CTC status and nodal status, as well as the weaker effects of vascular infiltration and histological type on survival of resected patients [20], we performed survival analyses subgrouped by these factors (Fig. 5). Patients with or without DTCs had similar outcome in those subgroups (Fig. 5). Presence other any other of the bone marrow ICC-positive cell types did not affect survival among the CTC-megative patients (Additional file 1). Among the CTC-positive patients, DTCs were not observed (Fig. 5) and in only one patient a single ICC-positive cell (HC) was detected (Additional file 1).

## Discussion

### DTC frequency

A low frequency of DTCs was detected in both resected (3.6%) and advanced cancer patients (6.5%). These numbers are less than half the frequencies reported from the largest patient series prior to the present study [13] which reported 13.5% DTC-positivity for resected cancers and 13.9% for advanced cancers. Even higher positive rates of 38 to 57.1% were reported from earlier cohorts with smaller patient numbers and predominantly advanced stage cancers [14, 15, 17]. Complementary to the data from ICC-based studies, there were two studies using RT-PCR for CK-20 [24] detecting 33% positive cases in a cohort of resectable PDAC [24] and CK-19 [25], detecting 67% positives in a cohort of mixed stage cases [25] (see also Table 3). In the absence of comparative studies between ICC and PCR-based detection, the seemingly higher positive rates for PCR-based assays targeting a single CK category versus ICC utilising broad spectrum antibodies against a range of class I and class II CK-subtypes remains unexplained, although different sensitivity and/or specificity may contribute.

### Survival prediction

Among resected pancreatic cancer patients, five ICC-based studies [13–17] reported a significant association between DTCs and impaired survival in univariate analysis (see also Table 3). In one of these studies [13], prognostic information was also retained in multivariate analysis (HR 2.755, *p* = 0.022), while the four other studies did not test independent predictive value of DTC. There are two additional ICC-based studies on the same subject that did not discover an association between DTC status and survival [26, 27]. When weighing the evidence from these studies, several methodological differences have to be taken into consideration. The ISHAGE-group [7] established the first consensus on the cytomorphological criteria for the classification of ICC-positive cells in the BM for breast cancer patients. These criteria were later underscored in consensus reports [8, 9] but also used in additional studies [23, 28].

Several of the studies mentioned above have been conducted prior to this standardisation or do not disclose their adherence to the ISHAGE criteria [14, 15, 17]. Furthermore, there is a lack of standardisation concerning the choice of CK-detection antibodies, only one group [27] used the same antibody combination as in the present study. While not validated separately for gastrointestinal cancer types, the broad reactivity of the AE1 and AE3 antibody combinations which covers a spectrum of both the basic and acidic categories of CKs makes the assumption reasonable that the assay employed in the present study detects CK-positive cells independent from the tissue-type dependent differences in CK-subtype expression. The comparison of our results with the aforementioned studies may also be affected by the small size of several of the studies and mixed cohorts with predominantly advanced cancer stages [14–17]. Moreover, the substantial rate of surgical interventions performed outside established criteria for operability may have introduced a hidden stage migration [13]. Concerning the association of UIC-type cells with an inferior OS in patients with benign disease, there are only two UIC-positive cases out of 30 patients, rendering the result inconclusive. Although there seemed to be an association of UIC with an improved DFS for UIC-positive patients in the resected group, no difference in overall survival was observed. Since DFS and OS are tightly linked in these patients the clinical relevance of this finding is questionable.

### ICC-positive cases in benign pancreatic disease

In the present study, ICC-positive cells meeting the morphological criteria for DTC were detected in four out of 29 patients with benign pancreatic disease, for one more patient the analysis was inconclusive. The majority of ICC-based studies have excluded patients with benign pancreatic disease a priori from the analysis [13–15, 17, 26]. Therefore, previous evidence on the validity of the results from the present cohort is limited. This observed rate of 13% is far above the established rate of 2–3.5% for false positive events for the ICC-based assay [7, 15, 27, 29]. The presence of occult malignancy in those patients as the source for those cells is unlikely since none of the patients were diagnosed with cancer later during the observation period and none had a previous history of malignancy. While the nature of those cells cannot be further determined in the present study, there are independent reports on cells of seemingly epithelial origin in the BM and peripheral blood of PDAC-patients. One ICC-based study reported results from four patients with benign disease, disclosing two ICC-positive cases [16]. In their conclusion the authors deem their method unfit for clinical use in PDAC due to the unreliability of the assay. Also one of the PCR-based studies report a rate of 11% in cases with benign pancreatic disease, while reporting only one positive case in 20 healthy controls [30]. In addition, there is one report disclosing the presence of epithelial cells in the bloodstream of patients with benign pancreatic disease [31]. These cells could potentially enter the BM via the bloodstream and could thereby be detected in BM-samples as well. Another possibility for the non-malignant origin of epithelial cells could be the shedding of epithelial cells from the pancreatic- and bile ducts or the duodenal wall during interventions, especially in the presence of inflammation [32]. Interestingly, all of the four ICC-positive cases in benign pancreatic disease in the present study had undergone invasive procedures preoperatively, either ERCP, endoscopic biopsy or bile duct stenting.

## Conclusions

The results from our study of a large cohort of patients with presumed resectable periampullary cancer show a low frequency of CK-positive cases in both resectable cancer, advanced cancer group and non-malignant pancreatic disease. No association with CK-status and survival was observed. For the UIC cell type, an association with disease free-survival was observed, but this was not supported by overall survival data and the relevance of the association is questionable. Cells satisfying predefined consensus criteria for DTC/CK-positive cells in the BM were detected both in patients with malignant as well as benign pancreatic disease. The combination of low frequency of CK-positive cells and their presence in benign pancreatic disease cases indicates that the ICC-based CK-detection method used in the present study does not reliably identify relevant cells of malignant origin for the type of cancers studied. Nevertheless, additional studies including further characterisation of the cells, preferably with single cell techniques are encouraged.

## Supplementary information

**Additional file 1 Figure S1:** Overall survival sub-grouped by CTC status in resected and advanced cancers according to non-malignant ICC-status in bone marrow. Legend: Overall survival dependent on CTC-status for resected and advanced cancer patients with (pos.) or without (neg.) ICC-positive cells. *P* values were computed by log-rank test assuming *p* < 0.05 for significance. The number of cases with determined CK-status differs from group size due to cases of inconclusive ICC-results.

**Additional file 2 Table S1:** Clinical parameters of all ICC-positive patients

## Data Availability

The datasets used and/or analysed during the current study are available from the corresponding author on reasonable request.

## Abbreviations

AJCC/UICC: American joint committee on cancer / union internationale contre le cancere
AMPUC: Ampullary cancer
APAAP: Alkaline phosphatase-anti-alkaline phosphatase
BM: Bone marrow
CDC: Centre for disease control
CI: Confidence interval
CK: Cytokeratin
CT: Computed tomography x-ray
CTC: Circulating tumour cells
DBDC: Distal bile duct cancer
DFS: Disease-free survival
DTC: Disseminated tumour cells
DUODC: Duodenal cancer
HC: Hematopoietic cells
ICC: Immuno-cytochemistry
ISHAGE: International society of hematotherapy and graft engineering
MNC: Mononuclear cells
OS: Overall survival
PDAC: Pancreatic ductal adenocarcinoma
pTNM: Pathological tumour stage, nodal status, metastastis status
QHC: Questionable/probable hematopoietic cells
REMARK: Reporting recommendations for tumour marker prognostic studies
STROBE: Strengthening the reporting of observational studies in epidemiology
TC: Tumour cells
UIC: Uninterpretable cells
