# Supplementary figures and images for: Cytokeratin-positive cells in the bone marrow from patients with pancreatic, periampullary malignancy and benign pancreatic disease show no prognostic information
Source: BMC Cancer. 2020 Nov 16;20:1107. doi: 10.1186/s12885-020-07510-z (PMC7667773; doi:10.1186/s12885-020-07510-z)

## Resected cancers

## Advanced cancers

UIC

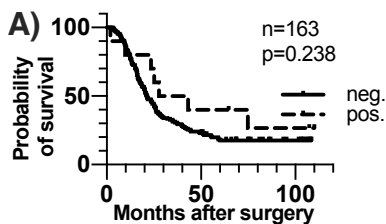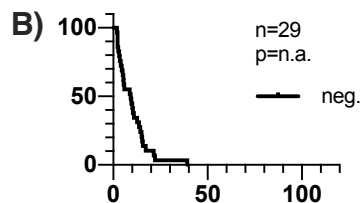

HC

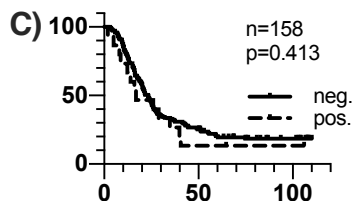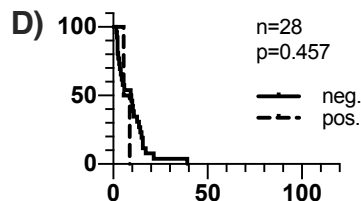

QHC

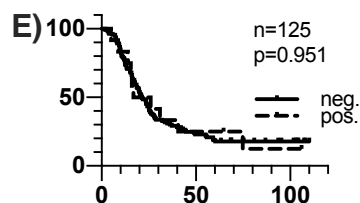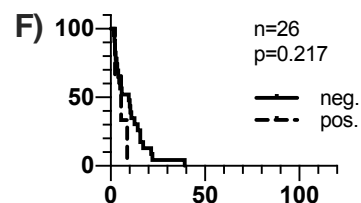

UIC

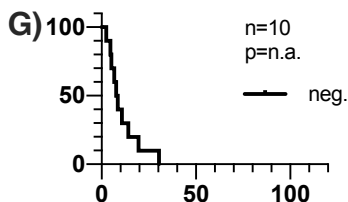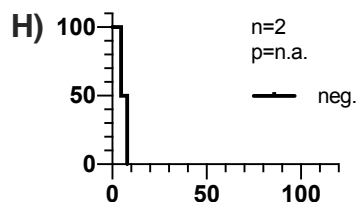

HC

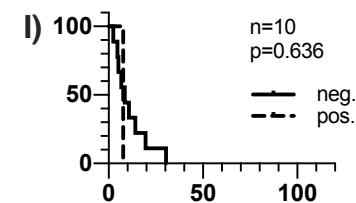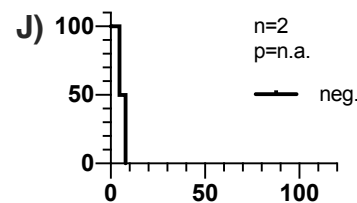

QHC

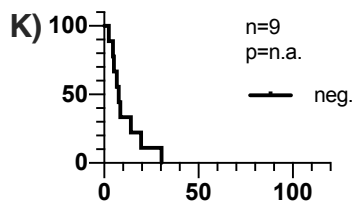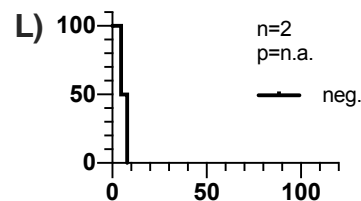

CTC-  
negative

CTC  
positive

Supplement: Supplementary file 1 — Additional file 1 Figure S1: Overall survival sub-grouped by CTC status in resected and advanced cancers according to non-malignant ICC-status in bone marrow. Legend: Overall survival dependent on CTC-status for resected and advanced cancer patients with (pos.) or without (neg.) ICC-positive cells. P values were computed by log-rank test assuming p < 0.05 for significance. The number of cases with determined CK-status differs from group size due to cases of inconclusive ICC-results. [file 12885_2020_7510_MOESM1_ESM.pdf]
