# Supplementary material for: Cytokeratin-positive cells in the bone marrow from patients with pancreatic, periampullary malignancy and benign pancreatic disease show no prognostic information
Source: BMC Cancer. 2020 Nov 16;20:1107. doi: 10.1186/s12885-020-07510-z (PMC7667773; doi:10.1186/s12885-020-07510-z)
Supplement: Supplementary file 2 — Additional file 2 Table S1: Clinical parameters of all ICC-positive patients [file 12885_2020_7510_MOESM2_ESM.docx]

Appendix Table 1: Clinical parameters of all ICC-positive patients

| Patient and tumour characteristics | | | | | | | | | | | | | **CK-positive cells** | | | | **Survival** | | |
| --- | --- | --- | --- | --- | --- | --- | --- | --- | --- | --- | --- | --- | --- | --- | --- | --- | --- | --- | --- |
| Age | **Cancer Type** | | **UICC-Stage** | | **pT** | | **pN** | **M** | **G** | **R** | **VI** | **PNI** | **TC** | **UIC** | **HC** | **QHC** | **OS [mo]** | **DFS [mo]** | |
|  | |  | |  | | **Benign disease** | | | | | | | | | | |  | |  |
| 82 | AIP | | St. 0 | | 0 | | 0 | 0 | 0 | 0 | 0 | 0 | **2** | **1** | 0 | n.e. | (62.1) |  | |
| 56 | INFLA | | St. 0 | | 0 | | 0 | 0 | 0 | 0 | 0 | 0 | **1** | 0 | **1** | 1 | (46.1) |  | |
| 75 | PAPAD | | St. 0 | | 0 | | 0 | 0 | 0 | 0 | 0 | 0 | **1** | 0 | 0 | n.e. | (76.5) |  | |
| 62 | IPMN | | St. 0 | | 0 | | 0 | 0 | 0 | 0 | 0 | 0 | **1** | 0 | n.e. | 0 | (71.5) |  | |
| 61 | CROP | | St. 0 | | 0 | | 0 | 0 | 0 | 0 | 0 | 0 | 0 | **1** | **1** | 3 | (81.0) |  | |
| *71* | *IPMN* | | *St. 0* | | *0* | | *0* | *0* | *0* | *0* | *0* | *0* | *0* | *0* | ***2*** | *1* | *(75.1)* |  | |
|  | |  | |  | | **Resected cancers** | | | | | | | | | | |  | |  |
| 69 | PANBIL | | St. Ib | | 2 | | 0 | 0 | 1 | 0 | 0 | 0 | **1** | 0 | n.e. | 0 | (101.8) | 49.5 | |
| 70 | INTEST | | St. IIb | | 1 | | 1 | 0 | 2 | 0 | 0 | 0 | **1** | 0 | 1 | 0 | 39.9 | 30.0 | |
| 60 | PANBIL | | St. III | | 4 | | 1 | 0 | 2 | 0 | 0 | 1 | **1** | 0 | 0 | **2** | 15.5 | 5.1 | |
| 64 | PANBIL | | St. IIb | | 3 | | 1 | 0 | 3 | 1 | 1 | 1 | **1** | 0 | n.e. | 0 | 26.1 | 13.3 | |
| 62 | PANBIL | | St. IIb | | 3 | | 1 | 0 | 2 | 0 | 1 | 1 | **1** | 0 | 0 | 0 | 28.3 | 11.7 | |
| 65 | PANBIL | | St. IIb | | 3 | | 0 | 0 | 3 | 1 | 1 | 1 | **1** | 0 | 0 | 0 | 5.9 | 2.8 | |
| 79 | INTEST | | St. III | | 4 | | 1 | 0 | 2 | 0 | 1 | 0 | 0 | **6** | n.e. | 0 | 25.1 | 25.1 | |
| 68 | INTEST | | St. IIb | | 2 | | 1 | 0 | 2 | 0 | 0 | 0 | 0 | **1** | **1** | **2** | (64.7) | (64.7) | |
| 73 | PANBIL | | St. IIb | | 3 | | 1 | 0 | 3 | 1 | 0 | 1 | 0 | **1** | **2** | 0 | 1.9 | 1.9 | |
| 64 | PANBIL | | St. IIb | | 3 | | 1 | 0 | 3 | 1 | 0 | 1 | 0 | **1** | 0 | **1** | 74.8 | 72.3 | |
| 60 | PANBIL | | St. IIa | | 3 | | 0 | 0 | 2 | 1 | 1 | 1 | 0 | **1** | 0 | n.e. | (110.2) | (110.2) | |
| 43 | PANBIL | | St. IIb | | 3 | | 1 | 0 | 3 | 0 | 0 | 1 | 0 | **1** | n.e. | 0 | (107.4) | 77.1 | |
| 60 | PANBIL | | St. IIb | | 3 | | 1 | 0 | 2 | 0 | 0 | 0 | 0 | **1** | 0 | n.e. | 43.1 | 36.0 | |
| 52 | PANBIL | | St. IIb | | 3 | | 1 | 0 | 2 | 0 | 1 | 1 | 0 | **1** | 0 | 0 | 23.5 | 23.5 | |
| 62 | PANBIL | | St. IIb | | 3 | | 1 | 0 | 2 | 1 | 0 | 1 | 0 | **1** | 0 | 0 | 9.7 | 6.1 | |
| 80 | PANBIL | | St. IIb | | 3 | | 0 | 0 | 2 | 0 | 0 | 1 | 0 | **1** | 0 | 0 | 27.7 | 25.0 | |
| *67* | *PANBIL* | | *St. IIa* | | *3* | | *0* | *0* | *3* | *0* | *1* | *1* | *0* | *0* | ***3*** | ***2*** | *40.6* | *10.2* | |
| *58* | *PANBIL* | | *St. IIa* | | *3* | | *0* | *0* | *2* | *1* | *1* | *1* | *0* | *0* | ***1*** | ***1*** | *5.0* | *3.0* | |
| *62* | *PANBIL* | | *St. IIb* | | *3* | | *1* | *0* | *2* | *0* | *0* | *1* | *0* | *0* | ***1*** | ***1*** | *13.9* | *11.7* | |
| *63* | *PANBIL* | | *St. IIb* | | *3* | | *1* | *0* | *3* | *0* | *1* | *1* | *0* | *0* | ***1*** | ***1*** | *16.9* | *4.8* | |
| *79* | *PANBIL* | | *St. IIb* | | *3* | | *1* | *0* | *2* | *1* | *1* | *0* | *0* | *0* | ***1*** | ***2*** | *8.7* | *5.9* | |
| *75* | *PANBIL* | | *St. IIb* | | *3* | | *0* | *0* | *2* | *1* | *1* | *1* | *0* | *0* | ***1*** | ***2*** | *26.1* | *22.4* | |
| *71* | *PANBIL* | | *St. IIb* | | *3* | | *1* | *0* | *2* | *0* | *1* | *0* | *0* | *0* | ***1*** | n.e. | *29.0* | *5.4* | |
| *71* | *PANBIL* | | *St. IIb* | | *3* | | *1* | *0* | *2* | *1* | *0* | *1* | *0* | *0* | ***1*** | n.e. | *7.8* | *2.6* | |
| *76* | *PANBIL* | | *St. IIb* | | *3* | | *1* | *0* | *2* | *1* | *1* | *1* | *0* | *0* | ***1*** | n.e. | *8.1* | *7.6* | |
| *78* | *PANBIL* | | *St. IIb* | | *3* | | *1* | *0* | *2* | *1* | *1* | *1* | *0* | *0* | ***1*** | *0* | *12.3* | *7.8* | |
| *77* | *INTEST* | | *St. Ib* | | *2* | | *0* | *0* | *2* | *0* | *1* | *0* | *0* | *0* | ***2*** | ***1*** | *(106.0)* | *(106.0)* | |
| *68* | *MUC* | | *St. IIb* | | *1* | | *2* | *0* | *0* | *2* | *1* | *1* | *0* | *0* | ***2*** | ***1*** | *16.0* | *9.8* | |
| *72* | *PANBIL* | | *St. IIb* | | *3* | | *1* | *0* | *2* | *0* | *0* | *1* | *0* | *0* | ***1*** | n.e. | *34.8* | *5.7* | |
|  | |  | |  | | **Advanced cancers** | | | | | | | | | | | | | |
| 53 | PANBIL | | St. III | | 4 | | 1 | 0 | 4 | 0 | 0 | 0 | **1** | 0 | **1** | **1** | 5.5 |  | |
| 80 | PANBIL | | St. IV | | 4 | | 1 | 1 | 3 | 0 | 2 | 2 | **2** | 0 | n.e. | 0 | 2.3 |  | |
| *74* | *PANBIL* | | *St. IV* | | *4* | | *1* | *1* | *3* | *0* | *0* | *0* | *0* | *0* | ***1*** | ***1*** | *8.8* |  | |
| *61* | *PANBIL* | | *St. IV* | | *4* | | *1* | *1* | *0* | *0* | *0* | *0* | *0* | *0* | *0* | ***1*** | *2.3* |  | |
| VI: vessel infiltration; PNI: neural infiltration; OS: overall survival; DFS: disease-free survival; AIP: autoimmune pancreatitis, INFLA: unspecific inflammation; PAPAD: papillary adenoma; CROP: chronic pancreatitis; IPMN intraductal pancreatic mucinous neoplasia; PANBIL: pancreaticobiliary type; INTEST: intestinal type; MUC: mucinous type; ( ): censored; n.e.: not evaluable, inconclusive analysis | | | | | | | | | | | | | | | | | | | |
